# Developing a research agenda for social prescribing in the UK using lessons from the US

**DOI:** 10.3399/bjgp22X721445

**Published:** 2022-11-25

**Authors:** Sahil Sandhu, Josephine M Wildman, Hugh Alderwick, John Wildman, Laura M Gottlieb

**Affiliations:** Harvard Medical School, Boston, Massachusetts, US.; Population Health Sciences Institute, Newcastle University, Newcastle upon Tyne, UK.; The Health Foundation, London, UK.; Business School, Newcastle University, Newcastle upon Tyne, UK.; Department of Family and Community Medicine, University of California San Francisco, San Francisco, California, US.

## INTRODUCTION

‘Social prescribing’ is a central component of NHS England’s long-term plan to provide more personalised care and reduce health inequalities. In social prescribing programmes, general practice staff refer patients to ‘link workers’ who connect patients with community-based services to address non-medical needs. In 2019, NHS England committed to a national rollout of social prescribing by funding a link worker for each of England’s 1300 Primary Care Networks — groups of general practices covering populations of around 30 000–50 000 people.^1^

Despite widespread policy support, research on the implementation and impact of social prescribing is limited and inconclusive.^2^ Many social prescribing evaluations have been of low methodological quality — with small numbers of participants, weak designs, no control groups, short durations, little consideration of confounding factors, and considerable loss to follow-up.^3^

In response to the weak evidence base, researchers have called for a coordinated evaluation framework to help develop a common body of knowledge on social prescribing.^3^ The National Academy of Social Prescribing in the UK launched an academic collaborative to define evidence gaps. The National Institute for Health and Care Research recently funded a multi-region evaluation of the national rollout in primary care.

As these efforts develop, researchers in the UK could learn from parallel efforts to evaluate social prescribing in the US. Healthcare systems in the US and UK vary widely in structure, cultures, and values — as do approaches to funding and delivering social services.

Nonetheless, like in the UK, efforts to identify and respond to patients’ social and economic conditions within the healthcare system have proliferated in the US.^4^ This may be down to a mix of factors, including growing awareness of the role of social factors in shaping health, and relatively low investment in social spending compared with other high-income countries. Regardless, forms of social prescribing are recommended by multiple US professional organisations, and have been incentivised by policymakers and payers. In 2019, the US National Academy of Medicine released a consensus report on integrating non-medical care into healthcare delivery that described the state of research, policy, and practice on social prescribing.^5^ The *American Journal of Preventive Medicine* also published a special issue on the evidence and evidence gaps on integrated health and social care in the US.^6^

In this article, we highlight major findings from US-based consensus and dissemination efforts on social prescribing research, and describe how the research agenda in the US might be used to inform similar research in the UK. Research could be strengthened in three areas: 1) social needs assessment, 2) intervention effectiveness, and 3) the sustainable implementation of effective programmes. Given that social prescribing involves a complex mix of interventions spanning multiple agencies and with impacts spread widely over space and time, a mix of methods will be needed to understand how these interventions are implemented in real-world settings, how they achieve their intended outcomes, for whom, and how this varies by context.

## RESEARCH ON SOCIAL NEEDS ASSESSMENT

In 2019, the National Academy of Medicine’s report included a framework outlining five healthcare system activities that could strengthen integration between health care and non-medical services: *Awareness*, *Adjustment*, *Assistance*, *Alignment*, and *Advocacy*.^5^ *Awareness* activities include strategies to identify patients’ social risks, such as food insecurity and interpersonal violence, and is considered the launching point for other interventions to address patients’ social needs.^5^ These assessments often involve patients completing a social needs questionnaire on a paper form or tablet before their visit with the healthcare provider. For example, the Protocol for Responding to and Assessing Patients’ Assets, Risks, and Experiences (PRAPARE) tool, used by nearly one-third of US community health centres, contains questions related to 27 demographic and social risk factors (Box 1). Health centres can streamline the tool to include questions most relevant to their own patient population and those for which they have protocols to address. An emerging area of mixed-methods research in the US is advancing understanding of both the psychometric validity and reliability of screening tools and their pragmatic validity.^7^

**Box 1.** Demographic and social risk domains in the PRAPARE screening tool

**Personal characteristics** Race/ethnicity Hispanic/Latino ethnicity Migrant/farmworker status Veteran status Limited English proficiency **Family and home** Household size Housing status Housing stability **Social and emotional health** Social integration/isolation Stress **Optional domains** Incarceration Refugee status Neighbourhood safety Interpersonal violence	**Money and resources** Education Employment Health insurance Income Food security Utilities security Clothing security Phone security Childcare security Medicine or healthcare security Other material security needs Transportation for medical needs Transportation for non-medical needs

*PRAPARE = Protocol for Responding to and Assessing Patients’ Assets, Risks, and Experiences.*

In contrast, social prescribing in the UK has focused less on standardised assessment of social needs. NHS England has not provided clear guidance to GPs on how to assess patients’ social needs and prioritise referrals to link workers. UK link workers are encouraged to assess patients holistically by asking what matters to them. But open-ended assessments may make it difficult to track population-level social needs, reasons for referrals, whether social prescribing interventions are reaching the right patients, gaps in community resources, and improvements in outcomes linked to different support. More systematic data on social needs and who is offered assistance could identify inequities in existing social prescribing schemes.

## EFFECTIVENESS RESEARCH

### Methodologically rigorous study designs

In the US, after patients are screened for social risks, providers might use this information to support care *Adjustment*, or activities to adapt care to accommodate social barriers to accessing health care (for example, increase telehealth visits for patients with transportation barriers). Additionally, providers might more directly help address patients’ underlying social needs by providing or referring patients to non-medical services provided by the clinic, community-based organisations (CBOs), or the government. Social prescribing interventions in the UK have not clearly distinguished between *Adjustment* and *Assistance* — though, in both countries, *Adjustment* activities have not been featured prominently in social prescribing research.

*Assistance* interventions in the US typically focus on connecting patients to resources that address basic material needs (for example, food, housing). The staff for assistance activities varies across health systems and may include traditional healthcare staff (for example, physicians, nurses, and medical assistants) or nonmedical staff (for example, social workers, community health workers, and volunteer navigators). In the UK, social prescribing programmes often involve GPs referring patients to link workers or other practice staff, who assess patients’ needs and refer them to a broader range of services (for example, exercise, arts and crafts activities, volunteering programmes) that support overall wellbeing and quality of life, alongside services for basic needs. This focus on wellbeing has resulted in ‘patient activation’ and ‘self-determination’ being prioritised in intervention development (for example, link worker coaching and motivational strategies), while many US approaches have emphasised signposting to community-based resources.^8^ Nonetheless, recent evidence from the UK suggests that accessing benefits related to housing, finances, and employment are major drivers of referrals to social prescribing services.^8^^,^^9^

In the US, a growing number of randomised controlled trials (RCTs) and quasi-experimental studies have examined the effect of *Assistance* programmes, with mixed results.^10^ To help address the need for more high-quality studies, the federal government funded a multi-site implementation with coordinated evaluation involving 29 sites across the country to evaluate the effectiveness of social prescribing. Participating sites are required to screen patients for social risks using a standardised tool. Patients are then randomised into a control group (who receive a list of resources tailored to the patients’ social needs) or to an intervention group (who are offered personal navigation services). Navigators often conduct an in-depth personal interview as part of a strengths-based assessment, use motivational interviewing techniques to create goals and action plans, refer patients to community-based or government resources, coordinate with other healthcare and community partners, and follow up with patients and troubleshoot barriers to resolving their needs. As of October 2020, 750 000 patients have been screened and 135 000 were eligible for navigation services.^11^ Preliminary data show patient acceptance of navigator support is high, but only 14% of patients had at least one social need resolved after navigation. The large-scale evaluation has been hampered by variability in implementation and COVID- 19, which spurred dramatic changes in social needs, and in service capacity and utilisation. But it is an example of what large national investment in evaluation of social prescribing could entail.

In contrast, lack of coordination has limited evaluation of social prescribing in the UK. Even at a local scale, no RCTs to determine the effectiveness of social prescribing have taken place in England since 2000.^2^ Mercer *et al* in Scotland recently conducted a cluster-randomised control trial of a social prescribing intervention and found no improvements in any health and wellbeing outcomes.^12^

Given that social prescribing programmes are complex interventions tailored to local context, RCTs can be difficult to conduct. However, the US has consistently demonstrated that RCTs are feasible.^2^ First, to ensure trial feasibility, core intervention components (for example, standardised staff training, templates for patient goal-setting activities) must be defined as much as possible. Second, collecting quantitative process measures can provide clarity on ‘active ingredients’ (for example, reason for referral, duration of intervention, link worker caseload, successful connections to referred community resources). Third, parallel process evaluations that leverage qualitative and mixed methods are critical to understand contextual factors and mechanisms shaping interventions. For example, in parallel to their community health worker RCT, US researchers conducted a qualitative process evaluation that triangulated chart abstraction, in-depth interviews, and participant observation to understand variation in patients’ response to the intervention.^13^

The national rollout of social prescribing link workers presents novel opportunities in the UK to support pragmatic trials (for example, stepped-wedge trials, implementation-effectiveness hybrid trials) and quasi-experimental study designs (for example, difference-in-differences studies, interrupted time series studies). Such approaches are starting to be used in the UK, with a recent quasi-experimental evaluation of a social prescribing scheme in North East England.^14^

## COMPARATIVE EFFECTIVENESS RESEARCH

Comparative effectiveness research is also being used in the US to inform health system decisions on social prescribing. An emerging area of research in the US is evaluating whether addressing some social needs (for example, housing) is more effective or cost-effective than addressing others (for example, transportation). For example, a Massachusetts study found that improved cardiovascular outcomes from one relatively light-touch social prescribing programme were more closely associated with transportation than food resources.^15^ In the UK, exploring which interventions are associated with improved outcomes could help prioritise healthcare activities.

Little is known about which patients benefit the most from social prescribing. In the US, there has been debate about whether social needs interventions should be targeted towards high-need, high-cost patients (that is, ‘superutilisers’), or to all patients as part of universal screening for social risks. NHS England has recommended that social prescribing be targeted towards patients with long-term conditions, patients with mental health conditions, patients with loneliness, and those with complex needs.^1^ Future studies in the UK might examine the varying effects social prescribing schemes might have on different patient populations and sub-groups.

Social needs interventions are diverse. In the US, few studies have compared intervention components, such as mode of social risk screening (for example, verbal, tablet, paper),^16^ timing of the intervention (for example, before, during, after the clinical encounter), intervention setting (for example, primary care, emergency care, inpatient care), the type of social prescriber (for example, doctors, nurses, social workers), or intensity of intervention (for example, one-time referral, consistent follow-up),^17^ all of which are likely to influence intervention effectiveness and cost-effectiveness. Identifying active intervention ingredients should be a priority for research in both countries.

## Impact on the voluntary sector

At the community-level, the *Alignment* and *Advocacy* activities in the US National Academy of Medicine framework include partnering with or investing in CBOs, organising cross-sector coalitions, or promoting policies that address unmet social needs. Another area of research in the US relates to the impacts of social prescribing on CBOs and the social service sector. Just as the NHS has funded link workers in primary care, the US government and private payers have created new financial flexibilities, incentives, and dedicated funding to enable health systems to assess patients’ social needs and connect them to social services.^4^ Unfortunately, these financial supports often do not extend to the community-based nonmedical services to which patients are referred. Studies have demonstrated that CBOs worry about their capacity to accept referrals from health care, that health care will ‘medicalise’ social services, and social prescribing will divert attention from much-needed upstream policies to address fundamental social conditions.^18^ As a result, social prescribing activities may unintentionally widen inequalities. Similar concerns exist in the UK.^4^ Qualitative studies could be used to understand perspectives of voluntary organisations on social prescribing and inform future partnerships.

## IMPLEMENTATION RESEARCH

There is also a need to study the implementation of social prescribing schemes and identify strategies that maximise uptake by clinical teams and patients.

### Patient engagement in social prescribing

Multiple studies in the US have shown that many patients experiencing social issues do not consistently express interest in related assistance from their healthcare provider, or do not attempt to access social supports after a referral.^7^ Reasons may include poor validity of screening tools, patient distrust or negative experiences with social services, patient perceptions about the role of the health system in addressing social needs, or patient needs being addressed elsewhere. A slate of recently published studies examine how different implementation strategies might increase patient interest in assistance.^19^ Recent US efforts have also highlighted the need to more explicitly design and tailor social prescribing interventions that are anti-racist to ensure that social prescribing does not inadvertently worsen health inequities for people of colour and people living in poverty. A recent review demonstrated that only 29% of 152 social prescribing studies included race or ethnicity in their analysis of effectiveness, and only 14% examined differential treatment effects by race.^20^ In parallel, UK researchers should consider mixed-methods approaches to evaluate patient engagement with social prescribing, with special attention to racial and minority ethnic groups. One opportunity is leveraging the public social prescribing observatory, developed by the University of Oxford and Royal College of General Practitioners, which visualises weekly data from over 1800 GP practices and enables stratification by ethnicity, region, age, indices of multiple deprivation quintiles, and gender.

### Technology for social prescribing

The US has seen a burgeoning investment in technology platforms that facilitate referrals from the health sector to CBOs.^4^ This and other electronic health record (EHR)-based innovations have presented opportunities to study the role of new technology in social prescribing. A recent clinical trial tested implementation of a technology platform that produces a personalised list of community resources from data in the patient EHR.^21^ Studying the feasibility and acceptability of digital tools in social prescribing may be an area of interest in the UK as these digital technologies become more widespread.

### Payment reform and quality measures’ influence on social prescribing

The shift from fee-for-service to value-based payment models in the US has created financial incentives to adopt interventions, such as social prescribing, which may improve population health and reduce costs.^4^ Some states are including social risk screening and/or implementation of social interventions as quality measures in payment contracts. Studies have started to explore the extent to which new payment models have affected the uptake of such interventions.^22^ Similarly, more research is needed to understand how NHS England’s policy of reimbursing link workers and financial incentives to increase referrals affects national-level adoption of social prescribing.^4^

## CONCLUSION

Social prescribing is growing in popularity among policymakers in the US and UK. However, policy support has far outpaced evidence on impact — and research on social prescribing now needs to catch up to inform future policy developments. Emerging efforts to coordinate social prescribing research in the UK can be informed by parallel efforts in the US, including research on social needs assessments, effectiveness of interventions, and factors shaping their implementation and sustainability.
